# CL3: Generalization of Contrastive Loss for Lifelong Learning

**DOI:** 10.3390/jimaging9120259

**Published:** 2023-11-23

**Authors:** Kaushik Roy, Christian Simon, Peyman Moghadam, Mehrtash Harandi

**Affiliations:** 1 Department of Electrical and Computer Systems Engineering, Faculty of Engineering, Monash University, Clayton, VIC 3800, Australia; mehrtash.harandi@monash.edu; 2 Data61, CSIRO, Brisbane, QLD 4069, Australia; peyman.moghadam@csiro.au; 3 School of Engineering, College of Engineering, Computing and Cybernetics, Australian National University, Canberra, ACT 2601, Australia; christian.simon@anu.edu.au; 4 School of Electrical Engineering and Robotics, Faculty of Engineering, Queensland University of Technology, Brisbane, QLD 4000, Australia

**Keywords:** lifelong learning, contrastive loss, catastrophic forgetting, class-incremental learning

## Abstract

Lifelong learning portrays learning gradually in nonstationary environments and emulates the process of human learning, which is efficient, robust, and able to learn new concepts incrementally from sequential experience. To equip neural networks with such a capability, one needs to overcome the problem of catastrophic forgetting, the phenomenon of forgetting past knowledge while learning new concepts. In this work, we propose a novel knowledge distillation algorithm that makes use of contrastive learning to help a neural network to preserve its past knowledge while learning from a series of tasks. Our proposed generalized form of contrastive distillation strategy tackles catastrophic forgetting of old knowledge, and minimizes semantic drift by maintaining a similar embedding space, as well as ensures compactness in feature distribution to accommodate novel tasks in a current model. Our comprehensive study shows that our method achieves improved performances in the challenging class-incremental, task-incremental, and domain-incremental learning for supervised scenarios.

## 1. Introduction

A neural network with **l**ife**l**ong **l**earning (L3) [1,2] capability must have two fundamental attributes: (i) an acquisition technique to learn new knowledge and fine-tune existing knowledge and (ii) a prevention mechanism to avoid severe interference on existing knowledge by a novel input. However, current deep neural networks (DNNs) have one crucial pitfall when attempting to learn novel concepts from a sequence of tasks; *the learned knowledge from previous tasks is highly influenced by novel tasks, resulting in a significant drop in performance when the DNN learns new tasks.* This phenomenon is often referred to as catastrophic forgetting [3,4,5].

To mitigate catastrophic forgetting, the use of a memory buffer is studied [6,7] in the literature. This family of L3 methods stores a subset of samples from previous tasks in a memory buffer and replays interleaved with new samples. However, memory-based approaches may become biased towards new tasks as the distinctiveness in feature representation decreases and the old feature space largely deviates due to a data imbalance issue [8]. Furthermore, the forgetting phenomenon is related to a more generic characteristic of neural networks, namely, stability–plasticity dilemma [9,10,11]. To tackle the stability–plasticity of neural networks, regularization (e.g., distillation)-based approaches [2,12,13] have also been studied. Regularization methods often impose constraints on updating the parameters of the DNN to retain previous knowledge. However, performance degradation on previous tasks in a challenging form of L3, namely, class-incremental scenario, has been observed [14]. In practice, distillation-based approaches feed both old and new models with samples from memory and apply constraint to ensure that the new model mimics the prediction of the old one for already-seen classes [6]. Instead of applying constraint on the probability space, feature-distillation-based approaches rely on maintaining a similar embedding space between an old and a new model [13,15,16]. For instance, PODnet [13] uses a Euclidean distance of an $L_{2}$-normalized feature vector by $L_{\mathrm{fd}}={∥z^{t-1}-z^{t}∥}^{2}$. The existing distillation loss (i.e., $L_{\mathrm{fd}}$) only compares a corresponding feature vector extracted from a sample using an old and new model, respectively, and might not be suitable for modeling a compact embedding space. Therefore, hybrid solutions that make use of a distillation loss (as a regularizer) along with memory have been developed [6,8,12,13], in the hope of aligning the weight space of DNNs.

Ideally, one wants to design a hybrid approach that is also capable of discriminating among feature representations belonging to different concepts/classes as new knowledge arrives. In this paper, we propose to use contrastive learning (CL) as a form of distillation to mitigate semantic drift in the feature distribution and catastrophic forgetting of old knowledge by shaping an embedding space in lifelong learning scenarios.

Contrastive learning has shown great success in encoding embedding spaces even in the absence of labels [17,18,19,20]. However, CL methods cannot readily be applied to L3 problems for tackling semantic drift and forgetting old knowledge since the standard contrastive loss only considers views generated using the current model and does not contrast the view from the previous model. Furthermore, there is no generalized form of CL for knowledge distillation in L3. Therefore a natural question to ask is, given the properties of CL, can CL be generalized using kernel methods to perform L3 with limited memory?

To address the catastrophic forgetting issues of memory-based L3, we propose an abstract form of CL, namely, CL3 that is a composition of view alignment, knowledge distillation, and distribution matching in a unified manner. CL3 encourages the model to minimize its discrepancy between positive pairs from new and old models while matching the latent representation to be uniform on a hypersphere.

Overall, our contributions in this paper are as follows:

- We present a generalized form of contrastive loss using the kernel method for contrastive knowledge distillation in an L3 scenario to ensure the robustness in latent space in a limited memory setting.
- Our proposed approach significantly improves the performance on MNIST, CIFAR-10, and Tiny ImageNet datasets in memory-based L3 scenarios.

### Related Work

In this section, we discuss the related regularization-based and replay-based lifelong learning methods.

**Regularization-based L3 methods:** Regularization methods alleviate catastrophic forgetting of prior knowledge by imposing constraint on the update of network parameters when learning a new task [21,22,23]. A knowledge distillation [24] strategy was first introduced to minimize the dissimilarity between an old task and a new one in learning without forgetting (LwF) [21] where the prediction of the current model is matched with old models’ prediction. PODnet [13] minimizes the discrepancies between an extracted feature vector using a new and an old model. Simon et al. in [25] proposed to model a feature space with a low-dimensional manifold for an old and a new model and minimized the distance between responses along geodesics connecting manifold. Synaptic intelligence (SI) [22] applied a regularization constrain on the gradient of the parameter updates. The elastic weight consolidation (EWC) [23] method used the diagonal of the Fisher information matrix as an importance measure for the weights to guide the gradient updates. Regularization approaches fail to retain old knowledge, and their performance degrades greatly when they are deployed in a class-incremental L3 scenario as they require to know the task-ID at an inference time, which is not available in class-incremental scenarios.
**Memory-replay-based L3 methods:** To address the limitation of LwF in class-incremental learning, iCaRL [6] used a fixed memory that stores the small sample sets that are close to the center of each class from old tasks and replayed the stored data with new tasks by applying knowledge distillation to retain the past information. The experience replay (ER) method [26] combined off-policy learning from memory and on-policy learning from novel dataset to maintain stability and plasticity, respectively. Aljundi et al. [27] formulated the replay memory sampling as a constrained optimization problem and used gradient information to maximize the diversity in replay memory. To improve the suboptimal performance of a random memory sample selection process, Aljundi et al. [28] proposed controlled memory sampling where they retrieved most interfered memory samples while replaying. An inherent dataset imbalance issue in memory-based L3 methods introduces bias in a neural network model when previous classes are visually similar to new classes. This bias in the last layer towards new classes was corrected to minimize forgetting in the BIC method [29] by employing a linear model with two parameters that is trained on a small validation set. Hou et al. [8] proposed a rebalancing method (LUCIR) to address the class imbalance issue by interclass separation, cosine normalization, and less-forget constraint. Instead of replaying raw samples, recent approaches [30,31,32] propose to replay low-dimensional latent feature.
**Generative-replay-based L3 methods:** Many recent approaches considered the lack of old samples as the reason for catastrophic forgetting, and instead of storing real samples, they addressed the problem by generating synthetic samples using an auxiliary network [30,33,34,35]. Deep generative replay (DGR) [33] proposed a two-model-based architecture, one for generating pseudo samples and another for solving tasks by replaying pseudo samples together with new samples. Generative feature replay (GFR) [30] replayed a latent feature instead of pseudo samples. However, a training generator network is troublesome, and a generator itself might experience chronic forgetting, which is not well investigated. Regardless of any pitfall, the supremacy of memory-based methods across the three scenarios of lifelong learning has been reported in [14,36].
**Contrastive-representation-learning based L3 methods:** Contrastive learning [17,37,38], a self-supervised learning [39,40] paradigm, has emerged as a powerful technique for representation learning. It learns representations by contrasting positive and negative samples and has proven effective in various tasks, including image classification [17,41,42], object detection [43,44], and natural language processing [45]. Consequently, contrastive representation learning has garnered substantial attention in recent years within the lifelong or continual learning literature [19,20,46,47,48,49,50]. By harnessing the principles of contrastive learning, L3 models can acquire representations that capture both task-specific information and general features. Varshney et al. in [49] proposed a lifelong intent detection framework that uses prompt augmented generative replay to generate new data for the current task by replaying data from previous tasks. It then augments these data with prompts and employs supervised contrastive learning to acquire representations through the contrast of positive and negative samples from the generated data. A contrastive vision transformer (CVT) [48] introduced a transformer architecture-based online continual learning framework that uses a focal contrastive learning strategy to achieve a better stability–plasticity trade-off. Supervised contrastive learning with an adaptive classification criterion for continual learning in [47] uses a contrastive loss to directly learn representations for different tasks, and a limited number of data samples are saved as the classification criterion. Cha et al. presented a rehearsal-based continual learning algorithm named Co${}^{2}$L in [20] that uses contrastive learning to learn and preserve representations continually. However, all of these L3 methods use a conventional contrastive loss function with a cosine similarity measure, which may not comprehensively represent the intricate relationships in the data. In contrast, our proposed CL3 method utilizes kernel methods (e.g., RBF kernel) as the similarity measure, allowing our method to learn nonlinear and complex relationships in the data. Furthermore, we introduce a kernel-method-based generalized form of contrastive loss for lifelong learning.

## 2. Materials and Methods

We begin this section by introducing some notations for L3. Let $T=\{T_{1},T_{2},\cdots ,T_{T}\}$ be a sequence of *T* tasks. In supervised L3, every task comprises a training set in the form

$$D_{t}={\left\{\left(X_{i},y_{i}\right)\right\}}_{i=1}^{n_{t}}.$$

Here, $X_{i}\in X_{t}\subset X$ denotes a data sample (e.g., an image of size W×H), and $y_{i}\in Y_{t}\subset Y$ is its associated target (e.g., label) at task *t*. The goal of L3 is to sequentially learn a model f:X→Y for each task at time *t* to map the inputs $X_{i}$ to their target outputs $y_{i}$ while maintaining the performance on all prior tasks (i.e., 1,2,⋯,t−1). We assume that a fixed-size memory M is available to store a subset of previously seen samples to mitigate catastrophic forgetting in L3.

In this paper, we are interested in three challenging forms of L3, namely, *class-incremental*, *task-incremental* and *domain-incremental* L3. In class-incremental L3, the learner is exposed to unseen classes sequentially (i.e., tasks constitute new unseen classes). Therefore, the set of labels in two distinct tasks are disjoint, $Y_{t}\cap Y_{t^{\prime }}=\emptyset \;;\;t\neq t^{\prime }$. In the domain-incremental scenario, the learner is presented with samples from different domains, but the label set is fixed (i.e., $Y_{1}=Y_{2}=\cdots =Y_{T}$). At the evaluation time, in both class-incremental and domain-incremental settings, the model should classify query samples from all classes observed during training, and no further information/guidance will be provided.

A principal way of addressing the problem of L3 is to realize a latent space and improve it progressively by observing new tasks while ensuring that the knowledge of prior tasks is maintained. Ideally, we would like the latent space to be discriminative enough, with samples of every class forming a compact and separated cluster from the rest. If such a space can be obtained in a sequential manner, then one can seamlessly perform L3 by designing a classifier acting on the latent space. Below, we describe our proposed L3 approach.

### 2.1. Contrastive Lifelong Learning

Due to the nature of the problem at hand (i.e., lack of task-ID), we make use of a DNN with two main modules, namely, a **(1) contrastive representation learning (CRL)** module and a **(2) classification** module. CRL is a feature extraction module that maps inputs to a shared and lower-dimensional latent space. In doing so, CRL aims to project similar samples onto the same regions in the latent space by forming and comparing positive/negative pairs. CRL, parameterized by Θ, has a fixed structure and updates its parameters to adapt to novel tasks. On the contrary, the classification module, as the name implies, realizes class-specific mappings and will grow in size upon seeing novel tasks.

### 2.2. Contrastive Representation Learning

The CRL module, parameterized by Θ, realizes a mapping in the form $f:X\to R^{n}$. Ideally, we would like the resulting latent space to be discriminative and representative of the tasks seen by the network. To achieve this, we propose to make use of contrastive learning [17,38,51]. The objective of contrastive learning is to make augmented views of the same example agree [17]. A widely used contrastive loss to encourage agreement is based on the cross entropy loss and can even be traced back to the seminal work of Goldberger et al. [52]. In short, given an augmented view of an example, the contrastive loss aims to classify a set of candidates into the positive example (i.e., the augmented views of similar examples belonging to the same class) and negative ones (augmented views belonging to a different class).

#### 2.2.1. Revisiting Contrastive Loss

Since we have access to annotated data in the supervised setting and have been inspired by [38], we propose to perform supervised contrastive learning when a task *t* is provided. Suppose a new task with data $D_{t}$ is provided, and let ${\left\{z_{i}\right\}}_{i=1}^{m}=f\left(\Psi \left(X_{i}\right);\Theta^{t}\right)$ be the *m* views of $X∋X_{i}\in D_{t}$ generated by a weak data augmentation method Ψ:X→X. Let $S(z_{i},z_{j})$ be a similarity function; the cross-entropy-based supervised contrastive loss for $X_{i}$ can be defined as

(1) $$\begin{array}{cc} \;L_{\mathrm{con}}\left(X_{i};\Theta^{t}\right) & :=\frac{1}{N}\;\;\;\sum_{j\in p_{\mathrm{pos}}\left(i\right)}\;\;\;\;\;-\log\frac{\exp\left(\mathrm{sim}\left(z_{i}^{t},z_{j}^{t}\right)/\tau \right)}{\sum_{k\in \{1\cdots K\}\setminus \{i\}}\;\;\;\;\;\;\;\;\exp\left(\mathrm{sim}\left(z_{i}^{t},z_{k}^{t}\right)/\tau \right)} \end{array}$$

(2) $$\begin{array}{cc} \; & \;:=-\overset{\mathrm{view}\;\mathrm{alignment}}{\overset{︷}{\alpha \sum_{j\in p_{\mathrm{pos}}^{t}\left(i\right)}\mathrm{sim}\left(z_{i}^{t},z_{j}^{t}\right)}}+\overset{\mathrm{distribution}}{\overset{︷}{\gamma \log(\sum_{k\in p_{\mathrm{neg}}^{t}\left(i\right)}\exp\left(\frac{\mathrm{sim}\left(z_{i}^{t},z_{k}^{t}\right)}{\tau }\right))}} \end{array}$$

(3) $$\begin{array}{cc} \; & :=L_{\mathrm{align}}\left(X_{i};\Theta^{t}\right)+L_{\mathrm{distrib}}\left(X_{i};\Theta^{t}\right)\;. \end{array}$$

Here, $S\left(v_{1},v_{2}\right)=\left({v_{1}}^{⊤}v_{2}\right)/(∥v_{1}∥∥v_{2}∥)$ refers to the cosine similarity between two projected feature vectors, $v_{1}\mathrm{and}\;v_{2}$ , and τ is a temperature value. K=mN denotes the total number of augmented views in a minibatch, provided that *N* and *m* are the batch size and the number of augmentations applied to a minibatch, respectively. $p_{\mathrm{pos}}\left(i\right)$ represents the set of indexes of all positive pairs for anchor *i* in the minibatch. α and γ are 1/Nτ and 1/N. Note that $L_{\mathrm{con}}\left(X\right)$ in Equation (1) is dependent on the set of labels (to form positive pairs) and works for single task in an offline training mode.

#### 2.2.2. Generalization of Contrastive Loss for L3

The standard contrastive loss based on cross-entropy can be decomposed as a combination of alignment and distribution terms [51]. However, the proposed abstract form is not suitable for the lifelong learning scenario where preserving knowledge (model’s weight) from previous tasks in the current model is crucial for alleviating catastrophic forgetting. The widely used approach to retain already-learned knowledge in the new model is via distillation. The conventional form of contrastive loss is not specifically designed for continual learning scenarios and has no specific mechanism to perform knowledge distillation. Therefore, we argue that the conventional form is not the best match for L3. To address these shortcomings, we first employ the old and new models in feature extraction for different views while making use of an augmentation method. Afterward, we decompose the contrastive loss into three terms, namely, view alignment, distillation, and joint distribution term. Here, the anchor view generated from the current model is aligned with all views belonging to the new task and memory independently. This matching of the anchor with views generated from the old model acts as a distillation term and helps to retain previous knowledge.

In case of consecutive tasks where each augmented view is fed into a current and old representation learner, $\Theta^{t}$ and $\Theta^{t-1}$, respectively, a positive pair can be represented as $P_{\mathrm{pos}}=P_{\mathrm{pos}}^{t}\cup P_{\mathrm{pos}}^{t-1}$ and $n=|P_{\mathrm{pos}}\left|=\right|P_{\mathrm{pos}}^{t}\left|+\right|P_{\mathrm{pos}}^{t-1}|=n^{t}+n^{t-1}$.

Considering this fact, to generalize the CL using kernels [53] for L3, assume that k:X×X→R is a kernel function. $K_{Z^{t}Z^{t-1}}$ represents the kernel gram matrix between $Z^{t}$ and $Z^{t-1}$. The $i^{th}$ row and $j^{th}$ column of $K_{Z^{t}Z^{t-1}}$ is $K_{Z^{t}Z^{t-1}}^{ij}k\left(z_{i}^{t},z_{j}^{t-1}\right)$, which is the kernel value between $z_{i}^{t}$ and $z_{j}^{t-1}$. Note that the cosine similarity, $S\left(z_{i},z_{j}\right)$, in the standard contrastive loss is a kernel function with normalized features. Therefore, assuming $k\left(z_{i},z_{j}\right)\frac{S(z_{i},z_{j})}{\tau }$, we represent the abstract form of contrastive loss for L3 as follows:

(4) $$\;\begin{array}{cc} L_{\mathrm{CL}3}(X_{i};\Theta^{t},\Theta^{t-1}):= & -\overset{\mathrm{view}\;\mathrm{alignment}}{\overset{︷}{\sum_{j\in P_{\mathrm{pos}}^{t}\left(i\right)}\;\;K_{Z^{t}Z^{t}}^{ij}}}-\overset{\mathrm{distillation}}{\overset{︷}{\sum_{j\in P_{\mathrm{pos}}^{t-1}\left(i\right)}\;\;K_{Z^{t}Z^{t-1}}^{ij}}}\\ \; & +\overset{\mathrm{joint}-\mathrm{distribution}}{\overset{︷}{\sum_{j\in P_{\mathrm{pos}}^{t}\left(i\right)}\log\left(\sum_{k\in P_{\mathrm{neg}}^{t}\left(i\right)}\exp\left(K_{Z^{t}Z^{t}}^{ik}\right)+\sum_{k\in P_{\mathrm{neg}}^{t-1}\left(i\right)}\exp\left(K_{Z^{t}Z^{t-1}}^{ik}\right)\right)}}\\ & :=L_{\mathrm{va}}\left(X_{i};\Theta^{t}\right)+L_{\mathrm{kd}}\left(X_{i};\Theta^{t},\Theta^{t-1}\right)+L_{\mathrm{jd}}\left(X_{i};\Theta^{t},\Theta^{t-1}\right)\;. \end{array}$$

The joint distribution term $L_{\mathrm{jd}}(X_{i})$ encourages the matching of the hidden representations to a uniform distribution on a hypersphere, as shown by Wang and Isola [54]. Therefore, and as discussed in [51], we can consider it as a form of distribution matching loss. The view alignment term $L_{\mathrm{va}}(X_{i})$ encourages the current model to learn consistent representation from multiple augmented views, while the distillation term $L_{\mathrm{kd}}(X_{i})$ aims to maximize the similarity between new representation and all positive pairs from old representations.

In our approach, we employ a radial basis function (RBF) kernel as the similarity measure. The RBF kernel excels in computing similarity by capturing linear and nonlinear data relationships. If $Z_{i}$ and $Z_{j}$ are the projected feature representations, an RBF kernel can be defined as

(5) $$\begin{array}{c} k\left(Z_{i},Z_{j}\right):=\exp\left(-\lambda ∥Z_{i}-Z_{j}{∥}^{2}\right);\lambda >0. \end{array}$$

Putting all together, to update CRL, we optimize

(6) $$L_{\mathrm{CRL}}\left\{\begin{array}{cc} E_{X∼D_{t}}L_{\mathrm{con}}\left(X;\Theta^{t}\right), & \mathrm{if}\;t=0\\ E_{X∼D_{t}\cup M}L_{\mathrm{con}}\left(X;\Theta^{t},\Theta^{t-1}\right), & \mathrm{otherwise} \end{array}\right.$$

**Figure 1:**
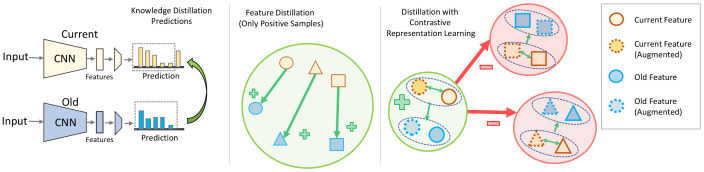
Distillation strategies: a comparison of knowledge distillation, feature distillation, and contrastive feature distillation. (**Left**) Knowledge distillation works on an output layer and matches the probability distribution between old and new models, (**middle**) feature distillation applies similarity constraints on the low-dimensional latent feature between prior and current models, and (**right**) contrastive feature distillation (ours), on the other hand, works on an even lower-dimensional projection space and minimizes distance between similar samples while maximizing distances between different samples.

Minimizing the loss in Equation (6) aims to (1) create a latent space where samples and their multiple views form compact and dense clusters, hence discriminative, and (2) increase the similarity between the latent representations for the model at time t−1 and time *t*. Figure 1 shows how our method differs compared with the previous methods by considering negative samples to minimize the distance of positive samples to learn a robust and compact latent space. In experiments, $L_{\mathrm{CL}3}$ outperforms standard $L_{\mathrm{CL}}$ by 3% and 5% on 10 tasks Tiny ImageNet with 200 exemplars for class-incremental and task-incremental settings, respectively.

The significance of CL3 regularization through distillation loss controls the updating of existing knowledge while adapting to new data, and it has been proven to be effective in mitigating catastrophic forgetting in the literature of L3. Knowledge distillation loss is often applied to the output layer, and a modified cross-entropy loss with temperature-scaled logit values is employed to map between the old and new probability distributions. Additionally, to preserve the structure of the embedding space, distillation on the feature space is also used by minimizing the distance between features extracted from the old and new models. However, the existing feature distillation losses only compare corresponding feature vectors extracted from a sample using the old and new models, respectively, and do not take advantage of the available label. Therefore, by leveraging contrastive learning, our proposed approach, $L_{\mathrm{CL}3}(X_{i})$, maximizes the similarity between all positive pairs among the old and new representations while also maximizing the distance between the negative examples as depicted in Figure 1.

The kernelized contrastive loss method holds paramount importance in lifelong learning as it enhances flexibility and expressiveness in measuring data sample similarity while learning a compact embedding space, where similar samples are mapped closely, and dissimilar samples are pushed farther apart. While traditional contrastive learning relies on basic similarity measures, such as Euclidean distance or cosine similarity, these may not sufficiently capture the intricate relationships within the data. In contrastive loss, kernel functions effectively replace the original similarity measures, providing a generalized kernel-based approach to contrastive learning. A prime example of such a method is the Gaussian radial basis function (RBF) kernel, which plays a pivotal role in uncovering complex nonlinear relationships within the data. The underlying idea with kernel functions is that they implicitly assess similarity by comparing feature representations within a higher-dimensional space. This approach is particularly valuable when dealing with intricate data distributions or when conventional similarity measures prove inadequate. In essence, kernelized contrastive learning offers a more adaptable and robust framework for measuring similarity, ultimately resulting in enhanced representation learning and improved performance within lifelong learning scenarios.

**Remark** **1** (form of data augmentation, Ψ). *Following a standard CL approach, we apply random augmentation: zero padding followed by random cropping and random horizontal flipping on an input sample to generate different views of it.*

**Remark** **2** (memory). *A small subset of previously observed samples is selected randomly and stored in memory. The contents of memory will be replayed along with novel samples to update the model. To reliably perform stochastic optimization, we will ensure that the number of samples per class is evenly distributed in a minibatch.*

### 2.3. Classifier

Once CRL is updated, to evaluate the performance of CRL, we optimize a linear layer, $\Phi^{t}$, as a classifier using standard cross-entropy loss:

(7) $$\begin{array}{c} L_{\mathrm{CLS}}\left(X_{i};\Phi^{t},\Theta^{t}\right)=-y_{i}\log\left(\Phi^{t}\left(\Theta^{t}\left(X_{i}\right)\right)\right)\;. \end{array}$$

Here, $\Phi^{t}\left(z_{i}\right)$ and $y_{i}$ are the prediction and corresponding target for sample $X_{i}\in \left(D_{t}\cup M\right)$.

## 3. Results

We begin this section by describing the datasets used in the experiments, implementation details, and training procedures. We then present the experimental results.

### 3.1. Datasets

We evaluate our proposed method, CL3, in three lifelong learning settings: class-incremental (CI), task-incremental (TI), and domain-incremental (DI). In our experiments, we use 3 different benchmark datasets: rotated MNIST [55] (R-MNIST), split CIFAR-10 [56] (S-CIFAR-10), and split Tiny ImageNet [57] (S-Tiny-ImageNet).

**MNIST** [55] consists of 70,000 grayscale images, each measuring 28×28 pixels, primarily showcasing handwritten digits spanning from 0 to 9. It is further categorized into a training set of 60,000 samples and a test set of 10,000 samples. **R-MNIST** [58] is a variation of the MNIST [55] dataset, wherein each task involves digits that have been rotated by a set angle between 0 and 180 degrees.
**CIFAR-10** [56] dataset consists of 60,000 color images, each with a resolution of 32×32 pixels. These images are classified into 10 distinct categories, with 6000 images per category, encompassing a diverse range of common objects, animals, and vehicles.
**Tiny ImageNet** [57] is a reduced-scale variant of the comprehensive ImageNet [59] dataset and provides a more approachable collection featuring 200 categories and a total of 100,000 images, all sized at 64×64 pixels.

To quantitatively analyze the performance of the L3 methods in CI and TI settings, we use S-CIFAR-10 and S-Tiny-ImageNet, which were created by splitting the CIFAR-10 and Tiny ImageNet datasets into 5 and 10 tasks, respectively. Each task in S-CIFAR-10 and S-Tiny-ImageNet consists of a nonoverlapping set of 2 and 20 classes, respectively. The splits of S-CIFAR-10 and S-Tiny-ImageNet are identical across different runs. We employ R-MNIST for DI setting, a dataset of 20 tasks created by rotating the original MNIST [55] images by a uniformly randomly chosen degree in [0,π).

### 3.2. Implementation and Training Details

We use ResNet18 [60] as the encoder to learn representations on S-CIFAR-10 and S-Tiny-ImageNet, following [12,20]. The representations are then mapped to a 128-dimensional latent space by a 2-layer MLP (projection module) with a hidden layer of 512 hidden units. For the R-MNIST dataset, we use a CNN with 3 layers as the backbone, consisting of two convolutional layers with 20 and 50 filters and a fully connected layer with 500 units. Additionally, a nonlinear projection head (2-layer MLP) with 500 neurons is employed for representation learning.

We utilize the data augmentation scheme from [17] in our training. We first crop the images in S-CIFAR-10, S-Tiny-ImageNet, and R-MNIST with scales of [0.2,1.0], [0.1,1.0], and [0.7,1.0], respectively, followed by resizing the cropped images to 32×32, 64×64, and 28×28, respectively. Additionally, we sequentially apply the augmentations *RandomHorizontalFlip*, *ColorJitter*, *RandomGrayScale*, and *GaussianBlur* with probabilities of 0.5, 0.8, 0.2, and 0.5, respectively, in S-CIFAR-10 and S-Tiny-ImageNet.

In our approach, for S-CIFAR-10 and S-Tiny-ImageNet datasets, the ResNet18 backbone is trained with a batch of 512 images for 500 epochs for the first task. However, for later tasks, the model is trained for 50 and 100 epochs on Tiny ImageNet and CIFAR-10, respectively. For the R-MNIST dataset, the backbone is optimized for 100 epochs during the first task and 20 epochs for later tasks. We use a stochastic gradient descent (SGD) optimizer with a momentum of 0.9 and a weight decay of 0.0001, along with a linear warmup for the first 10 epochs, followed by cosine decay for all experiments at every task for representation learning. However, for linear evaluation, we use SGD with a momentum 0.9 and a weight decay of 0 to train a linear classifier for 100 epochs. We decay the learning rate exponentially at 60, 75, and 90 epochs with a decay rate 0.2, and use learning rates of 0.01 for Seq-CIFAR-10, Seq-Tiny-ImageNet, and R-MNIST. Finally, we optimize our representation learner and classifier with a batch of 512 samples randomly selected from fixed-size replay memory and the current dataset in the supervised learning scenario. We use 0.2 as the temperature value across the experiments. We report the classifier’s test accuracy at the last task.

### 3.3. Experimental Results

We compare our proposed CL3 approach with state-of-the-art regularization-based (e.g., LwF [21] and oEWC [61]) and memory-based (e.g., iCaRL [6], AGEM [62], FDR [63], ER [26], DER [12], and DER++ [12]) lifelong learning methods. We also report the upper bound and lower bound of test accuracy, where the upper bound is trained with all observed tasks together and the lower bound does not employ any techniques to address catastrophic forgetting. The results for all baseline methods are extracted from [20]. LwF uses knowledge of a prior model to guide the current model to tackle forgetting. ER stores a subset of past samples to replay with novel samples. iCaRL extends LwF with herding-based memory exemplar selection and a nearest mean-of-exemplars classifier. FDR preserves network responses at the task borders to apply constraint on the change in network’s function space. A-GEM leverages memory samples to construct and enforce optimization constraints in the current updating process. DER and DER++ store logits together with exemplars in a memory buffer and use in a distillation process to preserve prior knowledge. Below we present a comparative analysis of L3 methods for class-incremental (CI), task-incremental (TI), and domain-incremental (DI) scenarios in the presence of a limited memory buffer. Overall, the results suggest that the memory-replay-based L3 methods (e.g., iCaRL, A-GEM, FDR, ER, DER, DER++, and CL3) outperform regularization-based methods (e.g., LwF, and oEWC) on both S-CIFAR-10 and S-Tiny-ImageNet datasets by a significant margin across settings.

**Figure 2:**
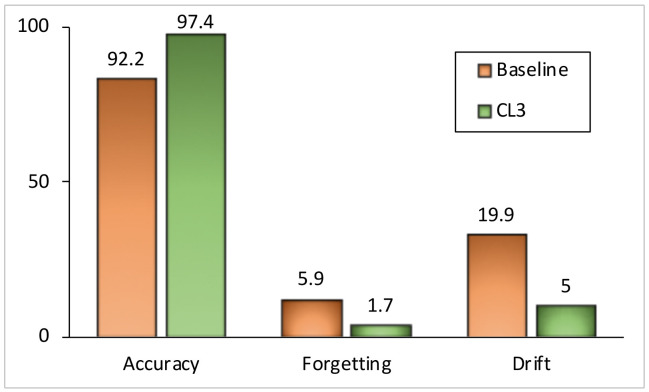
Comparative analysis of test accuracy, forgetting, and semantic drift: experience replay with knowledge distillation (baseline) vs. CL3 in a class-incremental scenario on a 5-task MNIST dataset with 5120 memory exemplars. CL3 shows significantly less drift on feature space and outperforms baseline by a significant margin in other metrics. Higher test accuracy, lower levels of forgetting, and semantic drift are better. Bar sizes have been adjusted for improved visualization.

**Class-Incremental (CI) Learning Scenario.** Table 1 represents classification accuracy on S-CIFAR-10 and S-Tiny-ImageNet datasets. In our experiments, regardless of the number of tasks across the datasets, regularization-based methods show a large drop in test accuracy as learning progresses. At the end of training on all tasks, we observe an unmatched performance gap between memory-based approaches and regularization-based approaches, particularly in the class incremental setting. The reason for this behavior is that those methods are specifically designed for the task-incremental learning scenario and require knowledge of the task identifier at the test time, which is not the case in the class-incremental learning scenario. In both datasets, our proposed method, CL3, outperforms other memory-replay-based methods. For example, CL3 exhibits around 20% and 5% improvement in performance compared with ER on S-CIFAR-10 and S-Tiny-ImageNet, respectively. CL3 performs comparatively with DER++ with 200 memory exemplars on S-CIFAR-10, while we note a roughly 2.3% better performance on S-Tiny-ImageNet. Furthermore, to investigate the applicability of CL3, we analyze the test accuracy, forgetting, and semantic drift [64] on five tasks’ MNIST data while storing 5120 exemplars in the memory buffer. As shown in Figure 2, CL3 can learn a more robust representation from a sequence of tasks, exhibiting less drift and forgetting while achieving higher accuracy compared with a vanilla end-to-end lifelong learning approach. For instance, we observed a 5% improvement in accuracy and 4% less forgetting using our proposed CL3 method compared with the experience replay method with knowledge distillation.

**Task-incremental (TI) learning scenario.** As reported in Table 1, all L3 methods perform significantly better in a TI setting compared with a CI setting because of the presence of a task identifier during the inference time. On an S-Tiny-ImageNet dataset, the CL3 method achieves comparable performance with other replay-based L3 methods. However, on an S-CIFAR-10 dataset, CL3 outperforms both regularization-based and memory-replay-based L3 methods.

**Domain-incremental (DI) learning scenario.** We also conducted a comparative analysis of L3 methods in a domain-incremental learning setting using the R-MNIST dataset with varying numbers of memory exemplars (200 and 500), as presented in Table 1. Across both memory settings, our proposed method, CL3, consistently outperforms its counterparts. For instance, when employing 200 memory exemplars, CL3 demonstrates an improvement of about 2% compared with the second-best-performing DER method. These results emphasize the potential of CL3 to enhance test accuracy in the context of domain-incremental learning, as demonstrated by the R-MNIST dataset.

**Figure 3:**
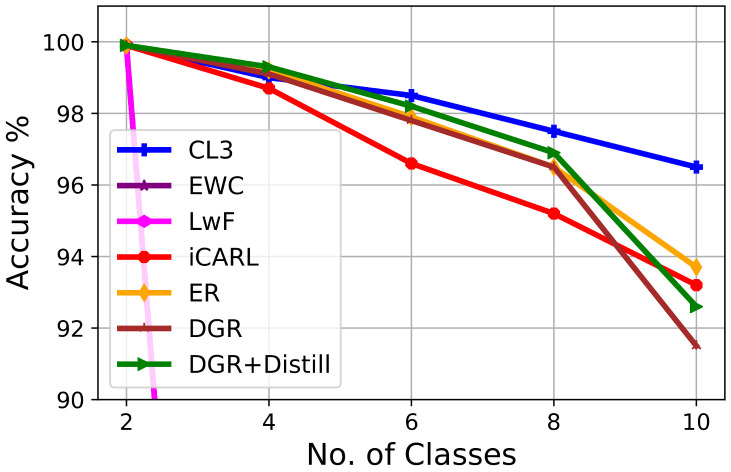
Classification accuracy evolution in class-incremental a 5-task MNIST dataset with a fixed memory of 200 exemplars. CL3 consistently demonstrates superior performance compared with other L3 methods across tasks.

Figure 3 depicts the gradual change in the performance of an L3 model while learning sequential tasks on a split MNIST dataset. In this experiment, we consider two regularization-based methods (e.g., LwF, EWC), two memory replay-based methods (e.g., iCaRL, ER), and two generative-replay-based methods (e.g., DGR, DGR+Distill). This figure suggests that CL3 exhibits consistent performances across the tasks and outperforms all other methods at every task. We observe that the performance of regularization-based approaches, e.g., LwF and EWC, drastically drops as learning progresses. In this setting, our method outperforms the state-of-the-art experience replay (ER) method by a margin of around 2% and iCaRL by 2.5% at the last task. The generative-replay-based method (DGR) performs comparably in the second task, and performance drops considerably along with other replay methods in the latter tasks, while CL3 shows consistent performance.

## 4. Discussion

In this section, we investigate the performance of our proposed contrastive lifelong learning method with (i) a different kernel method (ii) an increasing batch size, and (iii) a varying dimension of projection head.

**Figure 4:**
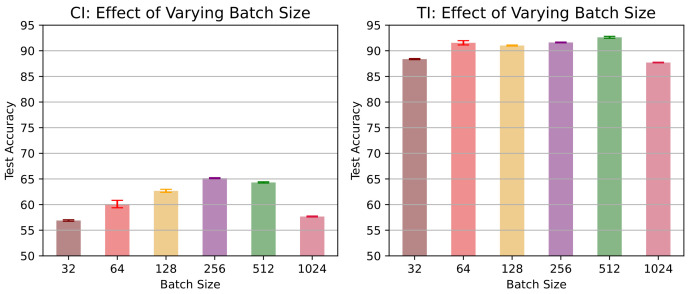
Classification accuracy in (**left**) class-incremental (CI) and (**right**) task-incremental (TI) scenarios for a 5-task CIFAR-10 dataset with a fixed memory of 200 exemplars and varied batch sizes. Different color bars represent varying batch sizes.

**Figure 5:**
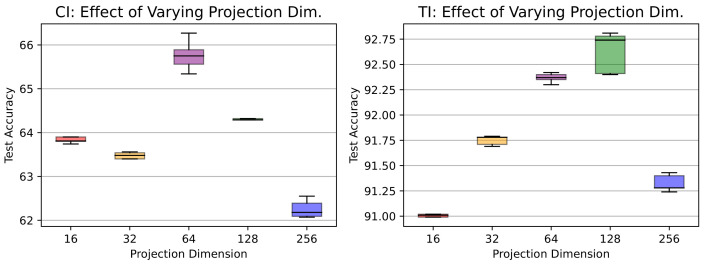
Classification accuracy on a 5-task CIFAR-10 dataset with varying projection dimensions for (**left**) class-incremental (CI) and (**right**) task-incremental (TI) settings. Different color boxes represent varying projection dimensions.

**Figure 6:**
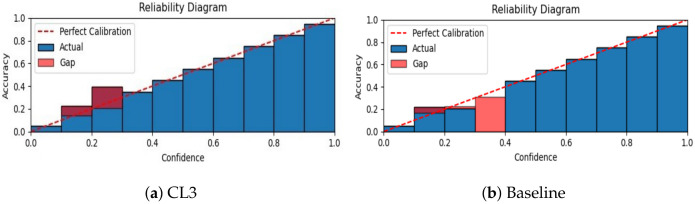
The calibration results for the class-incremental 5-task MNIST dataset with 200 exemplars in memory. Our proposed CL3 method outperforms the baseline, which is the experience replay method with knowledge distillation, in handling miscalibrated predictions.

**Efficacy of kernel method.** To assess the effectiveness of RBF kernel methods, we evaluate the performance of the cosine and RBF kernels across different datasets and memory settings in a class-incremental learning scenario and present the findings in Table 2. On the Tiny ImageNet dataset, both kernels exhibit comparable accuracy levels. However, the RBF kernel demonstrates marginal accuracy improvements. Conversely, on the CIFAR-10 dataset, the RBF kernel consistently outperforms the cosine kernel, achieving about 2% and 5% improved accuracy with a memory buffer of 100 and 200 exemplars, respectively. Overall, the results highlight the superiority of the RBF kernel over the cosine kernel in terms of accuracy, particularly across both CIFAR-10 and Tiny ImageNet datasets in a class-incremental learning scenario.
**Effects of increasing minibatch size.** To investigate the impact of varying minibatch sizes on the performance of CL3, we evaluated our proposed method using a 5-task CIFAR-10 dataset with a memory of 200 exemplars. The corresponding results are presented in Figure 4. The results suggest that in a task-incremental learning scenario, accuracy exhibited a positive correlation with larger batch sizes, reaching a peak at 512 and experiencing a slight drop at 1024. However, in a class-incremental learning setting, accuracy consistently increased as batch sizes expanded until it reached a peak at 256, followed by a period of stabilization and a slight decrease at 1024. This variation underscores that the batch size–accuracy relationship is context dependent.
**Effects of varying projection head sizes.** We also explore the influence of varying dimensions in the projection layer on CIFAR-10 and present the outcomes in Figure 5. The plot showcases the accuracy of a CL3 model under class-incremental (CI) and task-incremental (TI) learning settings with different projection head sizes. In CI, accuracy reaches a zenith of 65.7% at size 64, showing a minor dip at 256 (62.2%). Conversely, in TI, accuracy consistently advances with greater head size, achieving its highest point at 92.6% with a size of 128. These observations underline the dimension’s importance, revealing contextual differences.
**Calibration of neural network.** We calibrate the neural network’s predicted confidence values and visualize miscalibration using the reliability diagram presented in Figure 6. As depicted in the figure, our proposed CL3 method is more inclined to make accurate predictions, even when it is uncertain. Furthermore, the CL3 method demonstrates a lower number of incorrect predictions compared with the baseline method, even when it is highly confident. Overall, the reliability diagram clearly indicates that the CL3 method is more reliable than the baseline method.

## 5. Conclusions

In this paper, we introduce the contrastive L3 method, which consists of optimizing the feature encoder and learning a single-head linear layer classifier. Our method emphasizes learning low-dimensional, robust, and discriminative representations throughout the learning process by minimizing the distance between similar input samples and contrasting dissimilar samples from the replay buffer and the current task. Furthermore, we present the abstract form of the supervised contrastive loss as a combination of view alignment, feature distillation, and distribution mapping, making it suitable for the L3 scenario. In our experiments, we demonstrate that our proposed approach outperforms prior approaches for L3 in various settings, such as task-incremental, class-incremental, and domain-incremental. The superiority of our method shows that contrasting positive and negative samples for knowledge distillation yields an improvement to alleviate a well-known problem in L3, so-called *catastrophic forgetting*.

## Figures and Tables

**Table 1:** Test accuracy for L3 benchmarks on CIFAR-10, Tiny ImageNet, and R-MNIST. CI, TI, and Di refer to class-incremental, task-incremental, and domain-incremental learning setting, respectively. Best values are represented in bold.

Method	S-CIFAR-10		S-Tiny-ImageNet		R-MNIST	
**Setting**	**CI**	**TI**	**CI**	**TI**	**DI**	**DI**
Joint	92.20	98.31	59.8	82.04	98.67	
SGD	19.62	61.02	7.8	18.31	78.34	
LwF [21]	19.61	63.29	8.5	15.85	-	
oEWC [61]	19.49	68.29	7.58	19.20	-	
Memory M	200	200	200	200	200	500
iCaRL [6]	49.02	88.99	7.53	28.19	-	-
A-GEM [62]	20.04	83.88	8.07	22.77	89.03	89.04
FDR [63]	30.91	91.01	8.70	40.36	93.71	95.48
ER [26]	44.79	91.19	8.49	38.17	93.53	94.89
DER [12]	61.93	91.40	11.87	40.22	96.43	97.57
DER++ [12]	64.88	91.92	10.96	**40.87**	95.98	97.54
**Ours (CL3)**	**65.76**	**92.62**	**13.30**	39.83	**98.71**	**99.14**

**Table 2:** Comparative performance of cosine and RBF kernels as similarity metrics in CL3 on CIFAR-10 and Tiny ImageNet datasets with varied memory settings. RBF kernel consistently outperforms cosine similarity metric across settings. Best values are represented in bold.

Method	Kernel	CIFAR-10		Tiny ImageNet	
**Setting**		**CI (100)**	**CI (200)**	**CI (100)**	**CI (200)**
**CL3**	Cosine	50.72	60.49	10.93	12.51
**CL3**	RBF	**52.43**	**65.76**	**11.22**	**13.30**

## Data Availability

Our code will be publicly available at https://github.com/csiro-robotics/CL3. Publicly available datasets were analyzed in this study. This data can be found here: MNIST: http://yann.lecun.com/exdb/mnist/ (acessed on 30 October 2023) CIFAR10: https://www.cs.toronto.edu/~kriz/cifar.html (acessed on 30 October 2023) Tiny-Imagenet: http://cs231n.stanford.edu/tiny-imagenet-200.zip (acessed on 30 October 2023).
